# VE-cadherin interaction proteomics identifies ARVCF as stabilizer of endothelial adherens junctions

**DOI:** 10.1016/j.isci.2026.115450

**Published:** 2026-03-21

**Authors:** Rianne M. Schoon, Tsveta S. van Krimpen-Malinova, Iris de Heer, Arie J. Hoogendijk, Floris P.J. van Alphen, Annett de Haan, Anne-Marieke D. van Stalborch, Simon Tol, Jaap D. van Buul, Maartje van den Biggelaar, Stephan Huveneers

**Affiliations:** 1 Amsterdam UMC, University of Amsterdam, Department of Medical Biochemistry, Amsterdam Cardiovascular Sciences, 1105 AZ Amsterdam, the Netherlands; 2 Department of Molecular Hematology, Joint-AMC-Sanquin Landsteiner Laboratory, 1066 CX Amsterdam, the Netherlands; 3 Swammerdam Institute of Life Sciences, University of Amsterdam, Amsterdam 1098 XH, the Netherlands

**Keywords:** Vascular cell adhesion, Biochemistry, Cell biology

## Abstract

Blood vessel integrity is maintained by vascular endothelial-cadherin (VE-cadherin)-based adherens junctions, which form structural links between neighboring endothelial cells. In this study, we used mass spectrometry to identify the key proteins that interact with VE-cadherin. The proteomics identified a core group of proteins that bind to VE-cadherin, even when its intracellular domain is not tyrosine phosphorylated. The core VE-cadherin interactome includes known catenin proteins as well as ARVCF, ARHGAP23, KEAP1, and NGLY1. Co-immunoprecipitation and co-localization experiments verified that the VE-cadherin-binding protein ARVCF is a component of endothelial adherens junctions. During junction maturation, ARVCF selectively binds to a pool of VE-cadherin, which is unbound from p120-catenin, through a mechanism involving its C-terminal intrinsically disordered regions. Depletion of ARVCF results in unstable junctions, loss of endothelial barrier function, and impaired collective cell migration. Together, the results of this study demonstrate that ARVCF is an important stabilizer of VE-cadherin junctions to safeguard endothelial integrity.

## Highlights

• ARVCF is a component of endothelial adherens junctions
• ARVCF stabilizes endothelial cell-cell junctions
• ARVCF binds to a separate pool of VE-cadherin that is unbound from p120-catenin
• Proteomics identifies a robust VE-cadherin interactome

## Graphical abstract

**Figure:**
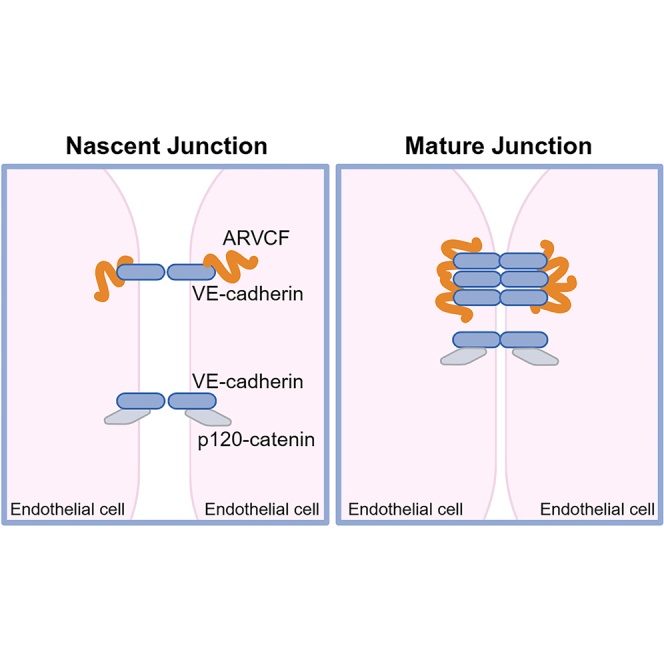

## Introduction

The luminal surface of arteries and veins is lined with a monolayer of vascular endothelial cells (ECs) that selectively allows for the exchange of cells and solutes between blood and tissue.^1^^,^^2^ This selective endothelial barrier is maintained through adherens junctions (AJ) formed through homotypic interactions of the transmembrane protein vascular endothelial-cadherin (VE-cadherin).^3^^,^^4^^,^^5^ The intracellular domain of VE-cadherin anchors to the actin cytoskeleton, which not only provides structural support but also allows for mechano-adaptive regulation.^6^ When VE-cadherin-based junctions are remodeled, for instance, by inflammatory cytokines, angiogenic growth factors, or chronic age-related alterations within the vessel wall, the endothelial barrier weakens, leading to (hyper)permeability, inflammation, and progression of cardiovascular diseases.^7^^,^^8^^,^^9^^,^^10^^,^^11^ The importance of a resilient endothelial barrier for vascular health is well established; however, the molecular mechanisms that safeguard the endothelial junctions are still incompletely understood.

The stability of endothelial AJs is intricately regulated by proteins that interact with VE-cadherin’s cytoplasmic domain. Classically, the AJ complex itself features direct and conserved VE-cadherin-binding proteins p120- and β-catenin, with the latter connecting via α-catenin to the actin cytoskeleton.^6^^,^^12^^,^^13^^,^^14^ In addition, other interacting proteins are known to play a role in endothelial barrier modulation.^15^ For example, tension across the VE-cadherin complex induces conformational change in α-catenin, exposing a binding site for vinculin whose presence stabilizes remodeling cell-cell contacts and fortifies attachment to the cytoskeleton network.^16^^,^^17^ Such force-induced junctional interactions are key for cell-to-cell communication and coordination across the endothelial tissue.^18^^,^^19^ Finally, phosphorylation and ubiquitination events at the cytoplasmic domain of VE-cadherin modulate its endocytosis, interaction partners, and association with the actin cytoskeleton, all of which are important for junction remodeling and barrier maintenance.^20^^,^^21^^,^^22^^,^^23^^,^^24^^,^^25^

VE-cadherin’s cytoplasmic domain contains three tyrosine (Y) residues at positions Y658, Y685, and Y731 that play a role in remodeling of the endothelial barrier.^26^ VE-cadherin is phosphorylated at Y658 and Y685, particularly in the venous side of the circulation.^8^^,^^27^ Phosphorylation of VE-cadherin at Y685 is further promoted by inflammatory cytokines, resulting in weaker AJs and vascular permeability.^8^^,^^28^ On the other hand, Y731 is constitutively phosphorylated in homeostatic endothelium, and leukocyte-endothelial interactions induce dephosphorylation of pY731, which is an essential event for leukocyte transendothelial migration.^7^ VE-cadherin-based AJs are, thus, tightly regulated through interacting proteins and post-translational modifications, which ensures both barrier function and selective permeability.

Here, we aim to elucidate whether the VE-cadherin complex is regulated through additional protein interactions. To study this, we employed a proteomics approach using mass spectrometry to determine the proteins that bind to VE-cadherin in comparison to VE-cadherin variants in which tyrosine residues were substituted for phenylalanine residues. Our results reveal a key set of previously unidentified proteins that bind to VE-cadherin. One of the identified core VE-cadherin complex proteins is armadillo repeat deleted in velo-cardio-facial syndrome (ARVCF), which strongly bound to all VE-cadherin variants. We demonstrate that ARVCF is crucial for endothelial barrier integrity of maturing monolayers, and that VE-cadherin-based adhesion stability is maintained by ARVCF through a unique mechanism that complements the function of other catenin-family proteins in the core AJ complex.

## Results

### Proteomics identifies VE-cadherin-binding proteins

**Figure 1:**
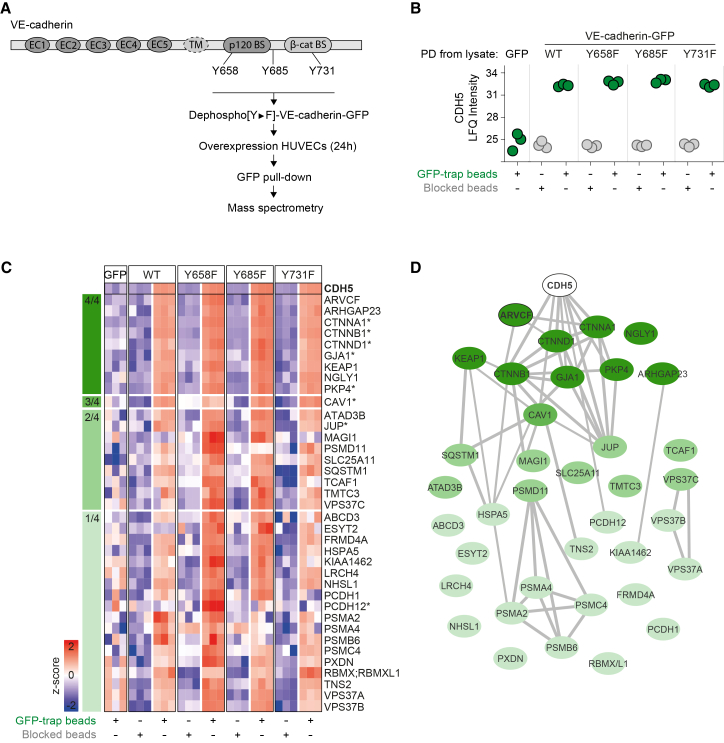
Identification of VE-cadherin-binding proteins (A) VE-cadherin contains five extracellular cadherin (EC) domains, a transmembrane (TM) region, and intracellular binding sites (BS) for p120-catenin and β-catenin. Workflow is indicated from generating and overexpressing VE-cadherin non-phosphorylatable [Y►F] variants in human umbilical vein endothelial cells (HUVECs) to mass spectrometry analysis. (B) Label-free quantification (LFQ) of VE-cadherin protein abundance detected by mass spectrometry of GFP pulldown (PD) samples versus blocked beads controls. The *y* axis indicates log_2_-transformed LFQ intensities; each dot represents a biological replicate. (C) Heatmap of significantly enriched VE-cadherin-binding proteins stratified by their interaction with wild-type and VE-cadherin Y►F variants. ∗ indicates previously reported VE-cadherin interactors. (D) STRING-based protein network analysis of detected VE-cadherin-binding proteins; lines indicate the interaction scores of >0.4. See also Figure S1.

To investigate protein interactions with VE-cadherin, we performed proteomic analysis on pulldowns of C-terminally GFP-tagged human VE-cadherin in human umbilical vein endothelial cells (HUVECs; hereafter referred to as EC). Mammalian VE-cadherin contains three tyrosine (Y) residues on the intracellular domain (Figure 1A), which are relevant for AJ stability and vascular integrity.^8^^,^^27^^,^^29^ We generated non-phosphorylatable VE-cadherin variants by substituting the cytoplasmic tyrosine (Y) residues 658, 685, and 731 by phenylalanine (F). The VE-cadherin-GFP variants were lentivirally expressed in ECs, which, in turn, were cultured to confluent monolayers. Next, we performed immunoprecipitations using magnetic GFP-trap beads. The obtained VE-cadherin pulldown samples were validated by western blotting by probing for the known interactor β-catenin,^30^ which confirmed successful specific co-immunoprecipitations (Figure S1A). Next, we performed label-free mass spectrometry on the GFP-pulldowns compared with blocked beads, showing equal levels of VE-cadherin detection of all variants (Figure 1B). The proteomic analysis, based on 3 independent IPs, with each IP derived from a separate transduction, identified 37 proteins that bind to VE-cadherin (Figures 1C and S1B). The mass spectrometry-based identification of the interaction partners of VE-cadherin and its non-phosphorylatable variants revealed a core VE-cadherin interactome, consisting of nine proteins, that is independent of Y phosphorylation of the cytoplasmic domain. The core VE-cadherin interactome includes the previously reported interactors p120-catenin (*CTNND1*), β-catenin (*CTNNB1*), α-catenin (*CTNNA1*), connexin-43 (*GJA1*), and plakophillin-4 (*PKP4*), and the identified ARVCF, ARHGAP23, KEAP1, and NGLY1 proteins. We detected 28 additional proteins, which displayed enriched binding to one or more of the VE-cadherin variants (Figure 1C). STRING analysis using predicted protein interactions indicated that ARVCF is closely linked to the functions of p120-catenin, β-catenin, and α-catenin (Figure 1D). Because ARVCF emerged as being previously unreported but as a strong binding partner of VE-cadherin, we decided to further investigate its interaction with VE-cadherin and function in ECs.

### Mechanism of the VE-cadherin-ARVCF interaction

**Figure 2:**
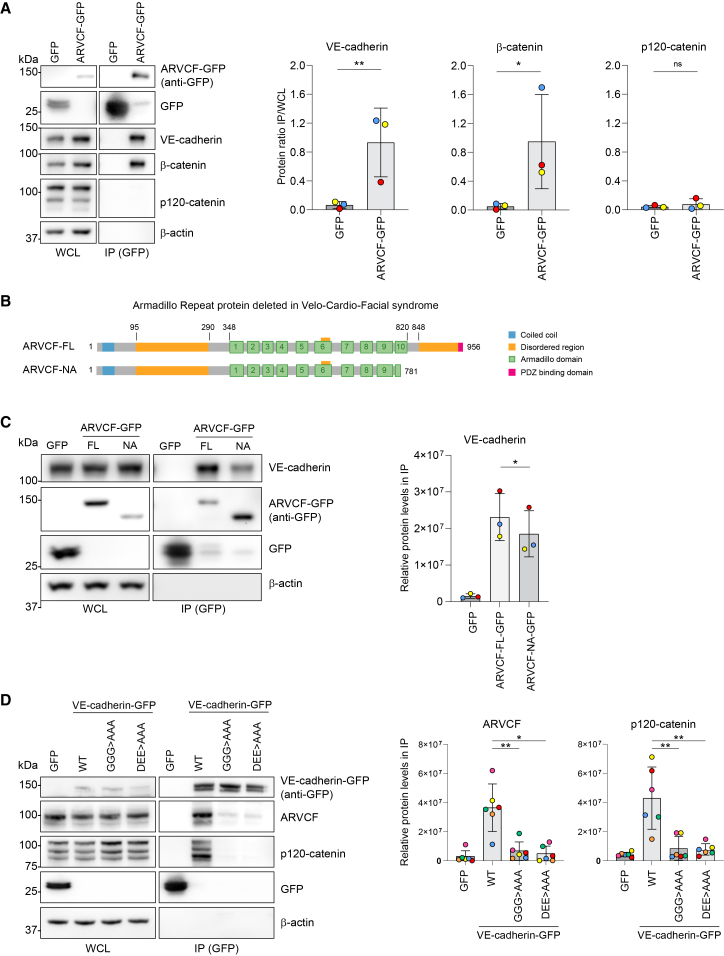
Mechanism of the VE-cadherin-ARVCF interaction (A) Representative western blot analysis probed for GFP, VE-cadherin, β-catenin, p120-catenin, and β-actin, on whole-cell lysates (WCLs) and GFP immunoprecipitation (IP) samples derived from HUVECs expressing GFP or GFP-ARVCF. Bar graphs show the ratio between IPs and WCLs of the quantified relative protein levels for VE-cadherin, β-catenin, and p120-catenin corrected for total protein loading using β-actin signal from three independent experiments. (B) Schematic overview of human ARVCF consensus protein, containing an N-terminal coiled-coil domain, a disordered region, 10 central armadillo repeats, and a C-terminal disordered region ending in a PDZ-binding motif. FL, full-length protein; NA, truncated protein containing only the N-terminal disordered region and the first 9 armadillo repeats. (C) Representative western blot analysis probed for GFP, VE-cadherin, and β-actin on WCL and IP samples derived from HUVECs expressing GFP, ARVCF-FL-GFP, or ARVCF-NA-GFP. Bar graph shows quantified VE-cadherin protein levels in GFP IPs corrected for total protein loading using WCL signal from three independent experiments. (D) Representative western blot analysis probed for GFP, ARVCF, p120-catenin, and β-actin on WCL and IP samples derived from HUVECs expressing GFP, VE-cadherin-GFP wild-type (WT), or its juxtamembrane domain mutants GGG>AAA or DEE>AAA. Bar graphs compare quantified ARVCF and p120-catenin protein levels in GFP IPs corrected for total protein loading using WCL signal from six independent experiments. The mean ± SD and values per independent experiment are shown. Student’s paired *t* test (C) or ratio paired *t* test (A and D); ∗*p* < 0.05; ∗∗*p* < 0.01; ns, non-significant. See also Figures S4 and S5.

To assess the interaction between VE-cadherin and ARVCF, we first generated a lentiviral construct expressing N-terminal GFP-tagged human ARVCF (canonical “long” isoform 1, UniProt: O00192-1, 962 amino acids). Immunoprecipitations of GFP-ARVCF from ECs resulted in robust levels of co-precipitated VE-cadherin, as well as the known classical cadherin-binding protein β-catenin, confirming the interactions that were detected by mass spectrometry (Figure 2A). Strikingly, ARVCF’s closest relative protein, p120-catenin,^31^ did not co-precipitate in GFP-ARVCF pulldowns (Figure 2A). The ARVCF protein contains an N-terminal coiled-coil domain, followed by a disordered region, central armadillo repeats, and a C-terminal disordered region ending in a PDZ-binding motif (Figure 2B). To investigate the molecular mechanism for binding to VE-cadherin, we compared C terminally GFP-tagged full-length ARVCF (UniProt: E9PDC3 isoform, 956 amino acids) with a truncated ARVCF variant lacking its C-terminal disordered region and PDZ-binding domain in immunoprecipitation assays. VE-cadherin co-immunoprecipitation from ECs was reduced in the absence of the ARVCF C-terminal domain (Figure 2C), indicating its importance for optimal binding to VE-cadherin. We next tested whether ARVCF interacts through the p120-catenin-binding domain in VE-cadherin by performing co-immunoprecipitations of ectopically expressed GFP-tagged wild-type VE-cadherin versus VE-cadherin^DEE>AAA^ or VE-cadherin^GGG>AAA^ variants, which lack a functional juxtamembrane domain.^32^^,^^33^ These experiments revealed that the VE-cadherin juxtamembrane domain is essential for ARVCF binding (Figure 2D). Together, these results indicate that ARVCF binds to the same domain in VE-cadherin as p120-catenin does, but it engages with a separate pool of VE-cadherin proteins that are unbound from p120-catenin.

### ARVCF localizes at VE-cadherin-based AJs

**Figure 3:**
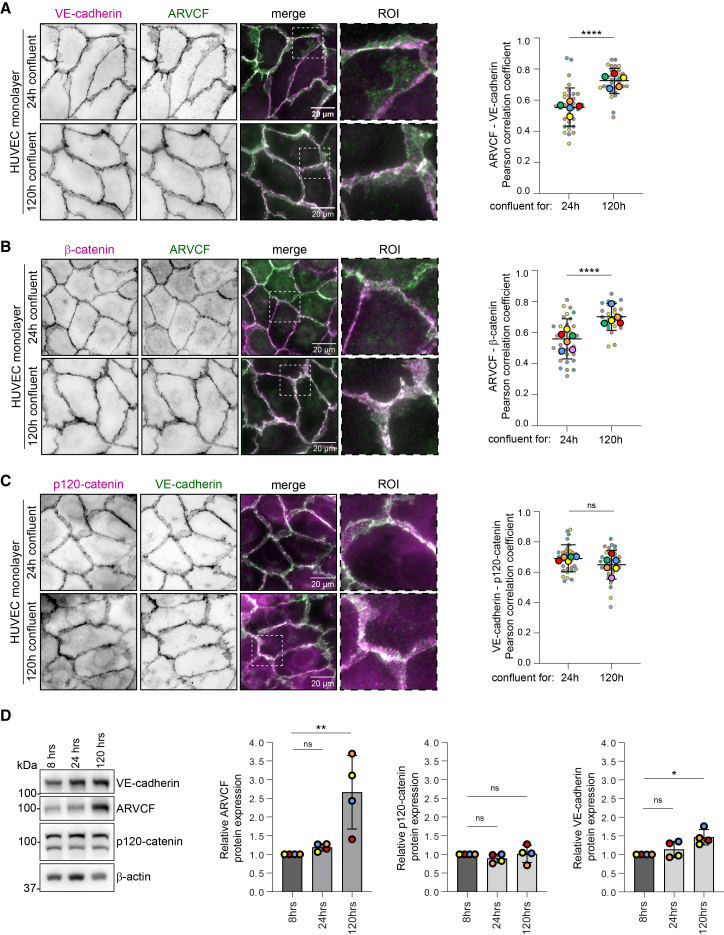
ARVCF localizes at VE-cadherin-based endothelial adherens junctions (A) Representative widefield images of HUVECs that were fixed after 24 or 120 h at full confluence and stained for endogenous VE-cadherin (magenta) and ARVCF (green). Bar graph showing Pearson’s correlation coefficients of pixel intensity values between ARVCF and VE-cadherin. Data are from *n* = 5 independent experiments, unpaired Welch’s *t* test. The graph shows values per image (small dots) and means from independent experiments (large dots per color) ±SD. (B) Representative widefield images of HUVECs that were fixed after 24 or 120 h at full confluence and stained for endogenous β-catenin (magenta) and ARVCF (green). Bar graph showing Pearson’s correlation coefficients of pixel intensity values between ARVCF and β-catenin. Data from *n* ≥ 5 independent experiments, unpaired Welch’s *t* test. The graph shows values per image (small dots) and means from independent experiments (large dots per color) ±SD. (C) Representative widefield images of HUVECs that were fixed after 24 or 120 h at full confluence and stained for endogenous p120-catenin (magenta) and VE-cadherin (green). Bar graph showing Pearson’s correlation coefficients of pixel intensity values between VE-cadherin and p120-catenin. Data from *n* ≥ 5 independent experiments; unpaired Welch’s *t* test. The graph shows values per image (small dots) and means from independent experiments (large dots per color) ±SD. (D) Representative western blot analysis of lysates derived from HUVECs after 8, 24, or 120 h at full confluence, probed for ARVCF, VE-cadherin, p120-catenin and β-actin. Bar graphs showing protein expression levels. Signal corrected for background, β-actin expression levels, and normalized to expression levels in the 8 h confluent condition. Data are from *n* = 4 independent experiments; ANOVA with Dunnett’s multiple comparison test. The bar graphs show mean ± SD and values per independent experiment. ∗*p* < 0.05, ∗∗*p* < 0.01, ns: non-significant. Scale bars are 20 μm. See also Figures S2 and S5.

ARVCF is a member of the catenin protein family, structurally related to α-catenin and β-catenin and most closely to p120-catenin.^31^^,^^34^ ARVCF has been reported to regulate AJs and tight junctions in epithelial cells.^35^^,^^36^^,^^37^ To gain insight into where ARVCF and VE-cadherin interact in ECs, we performed immunofluorescence assays to visualize endogenous ARVCF in endothelial monolayers. ARVCF was pronouncedly localized at cell-cell junctions in both venous and arterial ECs (Figures 3A and S2A). The localization of ARVCF and VE-cadherin differed slightly, resulting in junctional regions that were enriched for ARVCF (Figure 3A, see region of interest [ROI]). Notably, the level of co-localization between ARVCF and VE-cadherin increased substantially in mature junctions of 120 h confluent EC monolayers (Figure 3A). Similar results were obtained in immunofluorescence staining of ARVCF with β-catenin or α-catenin (Figures 3B and S2B). The co-localization between ARVCF and the AJ-independent junction protein PECAM-1 was unaffected by monolayer maturation (Figure S2C), suggesting a specific role for ARVCF at mature VE-cadherin-based AJs. Strikingly, and in contrast to ARVCF, the level of co-localization between VE-cadherin and p120-catenin did not change upon junction maturation in 120 h confluent monolayers (Figure 3C). At 24 h post-plating, subconfluent endothelial cultures exhibited reduced ARVCF and VE-cadherin co-localization compared with confluent cultures (Figure S2D). Western blot analysis of EC monolayers plated for 8, 24, and 120 h showed that the total ARVCF protein levels increased over time in confluency, whereas p120-catenin levels remained unchanged (Figure 3D). We next examined VE-cadherin and ARVCF co-localization during junction remodeling in thrombin-treated endothelial monolayers and found that ARVCF remained recruited to VE-cadherin-based junctions, with a slight, non-significant reduction (Figure S2E).

**Figure 4:**
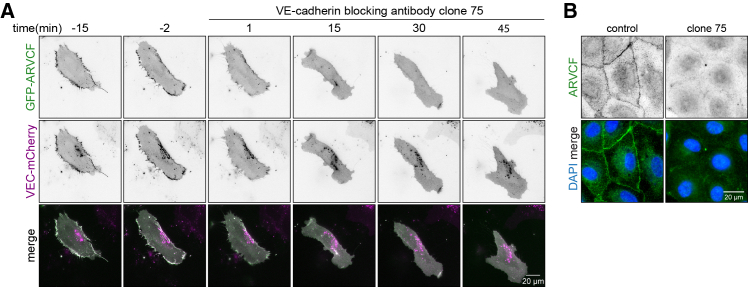
Junctional ARVCF localization depends on VE-cadherin (A) Stills from representative live imaging of HUVECs expressing both GFP-ARVCF (green) and VE-cadherin-mCherry (magenta) within an endothelial monolayer. VE-cadherin blocking antibody clone 75 (12.5 μg/mL) was added at t = 0, and cells were subsequently recorded for 1 h. (B) Representative widefield images of fixed HUVEC monolayers treated with 12.5 μg/mL VE-cadherin blocking antibody clone 75 for 1 h and immunostained for ARVCF and DAPI. Scale bars are 20 μm.

To investigate how ARVCF is recruited to junctions, we overexpressed GFP-ARVCF in ECs and subsequently inhibited the homotypic binding between VE-cadherin extracellular domains, using the clone 75 function-blocking antibody.^16^ The junctional localization of ARVCF was readily perturbed upon clone 75 treatment, showing that junctional ARVCF recruitment depends on VE-cadherin adhesion (Figures 4A and 4B; Video S1). Together, these experiments indicate that ARVCF-associated VE-cadherin-based adhesions are enhanced during junction maturation.

Video S1. Junctional ARVCF recruitment depends on VE-cadherin adhesion Related to Figure 4. Representative widefield movie of HUVECs overexpressing GFP-ARVCF and VE-cadherin-mCherry treated with 12.5 μg/mL VE-cadherin blocking antibody clone 75. Timescale, 30 s per frame.

### Depletion of ARVCF perturbs endothelial AJs and barrier function

**Figure 5:**
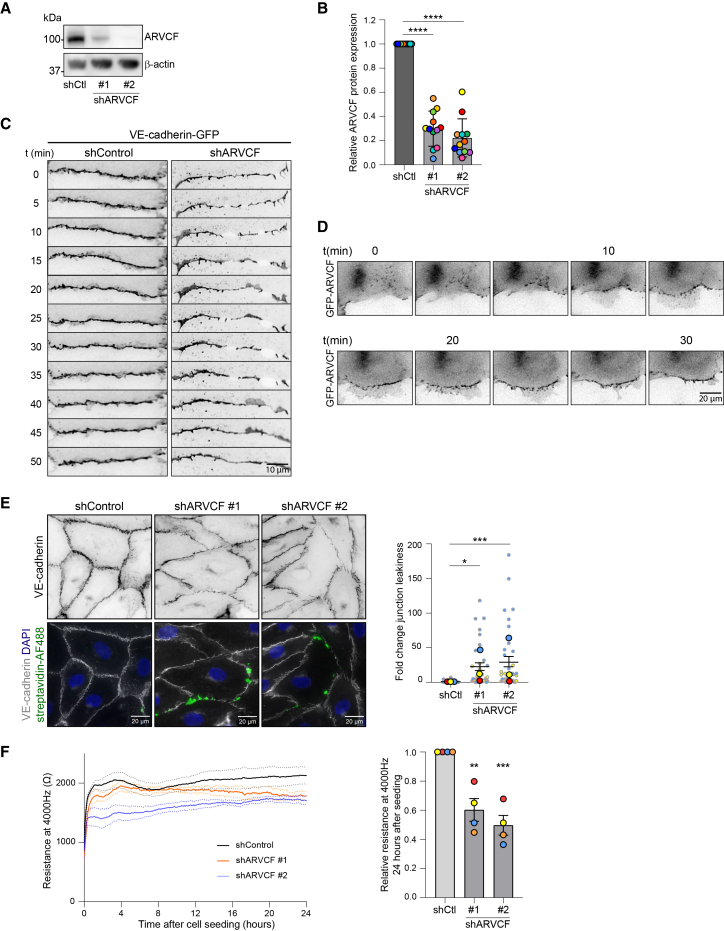
Depletion of ARVCF perturbs endothelial AJs and barrier function (A) Representative western blot analysis of lysates derived from HUVECs transduced with control or ARVCF shRNAs and probed for ARVCF and β-actin. (B) Bar graph showing the ARVCF protein expression levels. Signal corrected for background, β-actin expression levels, and normalized to expression levels in control cells. Data are from *n* = 12 independent experiments; ANOVA with Dunnett’s multiple comparison test was used. The mean ± SD and values per independent experiment are shown. (C) Representative stills from time-lapse widefield imaging every 5 min for 1 h of live HUVECs transduced with shControl or shARVCF and VE-cadherin-GFP. (D) Representative stills from time-lapse imaging every 10 min of live HUVECs transduced with GFP-ARVCF. (E) Representative images of fixed shControl and shARVCF HUVECs grown on biotin-gelatin and stained for endogenous VE-cadherin (gray), nucleus (blue), and streptavidin-AlexaFluor488 (green). Bar graph shows the fold change in junction leakiness between shControl and shARVCF HUVECs. Data are from *n* = 3 independent experiments; ANOVA with Dunnett’s multiple comparison test was used. The graph shows values per image (small dots) and means from independent experiments (large dots per color) ±SEM. (F) Line graph showing the average resistance (±SEM, dotted lines) measured with ECIS at 4,000 Hz of shControl and shARVCF HUVEC monolayers over time. Data are from *n* = 4 independent experiments. Bar graph showing the relative resistance of shARVCF monolayers at 4,000 Hz, compared with shControl cells, 24 h after cell seeding. Data are from *n* = 4 independent experiments; ANOVA with Dunnett’s multiple comparison test was used. The bar graph shows the mean ± SEM and values per independent experiments. ∗*p* < 0.05, ∗∗*p* < 0.01, ∗∗∗*p* < 0.001, ∗∗∗∗*p* < 0.0001. Scale bars are 10 or 20 μm. See also Figures S4 and S5.

To assess the importance of ARVCF in EC-cell junctions, we silenced *ARVCF* by using two independent lentiviral short hairpin RNAs (shRNAs), both of which induced efficient knockdown levels (Figures 5A and 5B). Flow cytometry analysis showed that VE-cadherin surface expression was maintained in ARVCF-knockdown cells, with levels comparable to those in shControl cells (Figure S3A). Immunofluorescence imaging confirmed that ARVCF-knockdown cells still formed VE-cadherin-based AJs, with an overall fluorescence intensity comparable to that of shControl (Figure S3B). Interestingly, the junction organization was altered in shARVCF ECs, with disrupted adhesions at cell-cell contact interfaces, resulting in non-continuous VE-cadherin distribution between ECs (Figure S3B). To understand how ARVCF controls junctions, we next investigated the dynamics of VE-cadherin by lentiviral overexpression of VE-cadherin-GFP in shControl and shARVCF ECs. Subsequent live-cell imaging experiments showed that the junctional interface between ARVCF-depleted cells was highly unstable, going through repeated cycles of junction formation and breakdown without junctional stabilization. This led to the formation of intercellular gaps along junctions over time (Figure 5C; Video S2). Live imaging of GFP-ARVCF in ECs further showed that ARVCF was recruited to the junctions in a punctate pattern during nascent junction formation (Figure 5D, top row; Video S3). After initial cell-cell contact formation, and when junctions were stabilized through cell protrusions, ARVCF was redistributed in a continuous, uninterrupted pattern between adjoining cells (Figure 5D, lower row; Video S3). Hence, the majority of ARVCF is recruited during cell-cell junction maturation, fitting with its role in junctional stabilization. To test whether ARVCF is necessary for stable cell-cell contacts, we assessed the integrity of endothelial junctions by culturing the ECs on biotin-coated gelatin substrates. Once confluent monolayers were established, fluorescent streptavidin was added to the apical side of the monolayers for 10 min to determine the level of junction leakiness. These experiments indicate that the depletion of ARVCF induces a considerable increase in intercellular gaps and leads to monolayer leakiness (Figure 5E). To investigate the importance of ARVCF for endothelial barrier function, we next used electrical cell-substrate impedance sensing (ECIS) measurements at 4,000 Hz across shControl and shARVCF EC cultures. These experiments showed that shARVCF cells had a significantly reduced capacity to form stable endothelial barriers, resulting in impaired endothelial barrier function over time (Figure 5F). We conclude that ARVCF is necessary for endothelial junction stabilization and monolayer barrier formation.

Video S2. Depletion of ARVCF perturbs endothelial AJs Related to Figure 5. Representative widefield movies of shControl and shARVCF transduced HUVECs overexpressing VE-cadherin-GFP. Timescale, 30 s per frame.

Video S3. ARVCF recruitment during AJ maturation Related to Figure 5. Representative widefield movies of HUVECs overexpressing GFP-ARVCF. Timescale, 30 s per frame.

### Junctional reinforcement restores endothelial integrity in the absence of ARVCF

**Figure 6:**
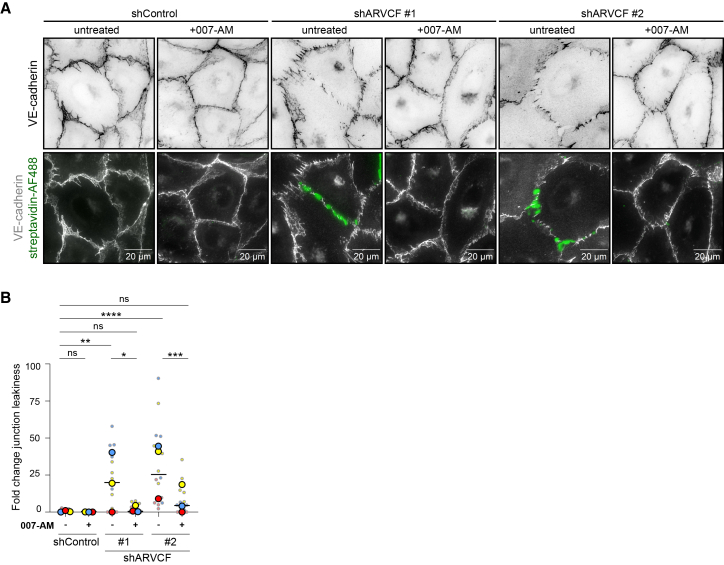
Junctional reinforcement restores endothelial integrity in the absence of ARVCF (A) Representative images of fixed HUVECs grown on biotin-gelatin, untreated or treated with 1 μM 007-AM for 10 min, and stained for endogenous VE-cadherin (gray) and streptavidin-AlexaFluor488 (green). (B) Bar graph showing the mean fold change in junction leakiness between shControl and shARVCF HUVECs with and without 007-AM treatment. Data are from *n* = 3 independent experiments; ANOVA with Tukey’s multiple comparison test was used. The bar graph shows values per image (small dots) and means per independent experiment (large dots per color). ∗*p* < 0.05; ∗∗*p* < 0.01; ∗∗∗*p* < 0.001; ∗∗∗∗*p* < 0.0001; ns, non-significant. Scale bars are 20 μm.

Previous studies on other tissues have shown that ARVCF controls intracellular force production by linking cell-cell adhesions.^38^^,^^39^ As such, ARVCF has been shown to regulate tension on junctions during xenopus embryonal axis elongation.^38^ To investigate whether the leaky junctions between ARVCF-depleted ECs were caused by a lack of junctional reinforcement, shControl and shARVCF ECs were treated with 8-(4-chlorophenylthio)-2′-*O*-methyladenosine 3′,5′-cyclic monophosphate (also known as 007-AM), a selective EPAC1 agonist that tightens the endothelial barrier through Rap1-mediated junctional reinforcement.^40^ After 10 min of 1 μM 007-AM treatment, ECs adopted hexagonal organizations within monolayers in all conditions (Figure 6A), which is indicative of an optimal distribution of radial forces and junctional tension in support of junction stabilization.^41^^,^^42^^,^^43^ Intriguingly, 007-AM fully restored the intercellular gaps between shARVCF ECs, thereby reducing junction leakiness (Figures 6A and 6B). These results point to a role for ARVCF in junctional reinforcement for endothelial barrier function.

### ARVCF is needed for collective cell migration

**Figure 7:**
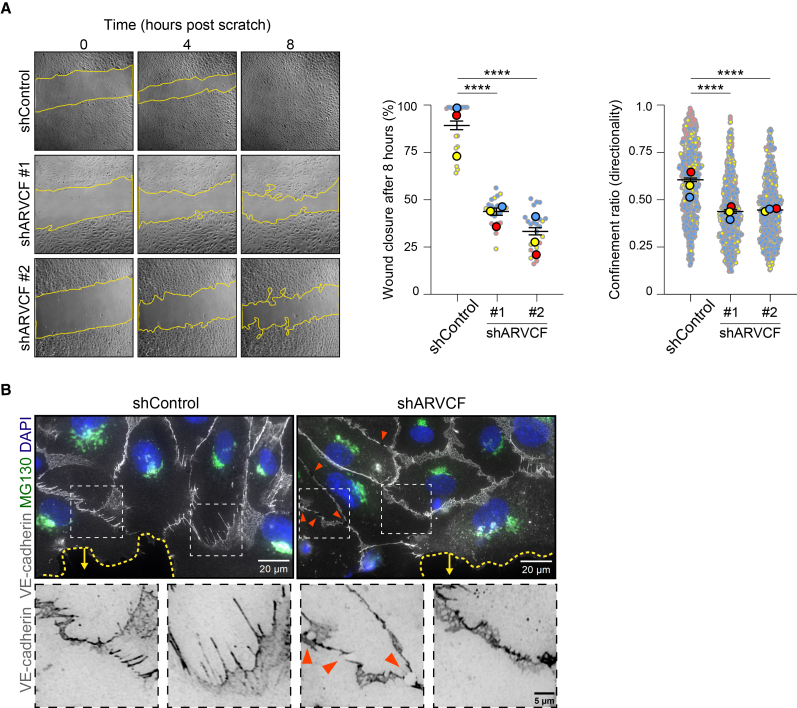
ARVCF coordinates collective cell migration (A) Representative images of HUVECs transduced with shControl or shARVCF in scratch wound assays. Wound closure upon scraping of confluent monolayers was tracked every 15 min for 10 h total. Left graph shows wound closure after 8 h in percentage of shControl and shARVCF cells. Values per scratch wound (small dots) and means from independent experiments (large dots per color) ±SEM are shown. Right graph shows the confinement ratio over 8 h of wound closure of shControl and shARVCF cells. Values per cell (small dots) and means from independent experiments (large dots per color) ±SEM are shown. Data are from *n* = 3 independent experiments, ANOVA with Dunnett’s multiple comparison test was used. ∗∗∗∗*p* < 0.0001. (B) Representative images of HUVECs transduced with shControl or shARVCF in scratch wound assays stained for endogenous VE-cadherin (gray), Golgi (green), and nucleus (blue). Orange arrowheads indicate AJ disruptions. Scale bars are 5 or 20 μm.

Endothelial junctions integrate mechanical cues derived from the pushing and pulling between cells to coordinate collective cell migration.^44^^,^^45^ To assess the importance of ARVCF for collective EC migration, we performed scratch wound assays in shControl and shARVCF monolayers. The depletion of ARVCF strongly inhibited migration during wound healing (Figure 7A). The defect in collective cell migration was related to an intrinsic lack of coordinated directionality between the migrating shARVCF cells (Figure 7A). Moreover, intercellular gaps appeared between the migrating leader and follower shARVCF cells (Figure 7B). These results show that ARVCF is important for junction remodeling during collective cell migration.

## Discussion

VE-cadherin is central to the build-up of endothelial AJs, yet the molecular diversity of its composition and its regulation remain incompletely understood. In this study, we used a proteomics approach through which four core VE-cadherin interactors were identified: ARVCF, ARHGAP23, KEAP1, and NGLY1. Co-immunoprecipitation and co-localization experiments verified that ARVCF is an important component of endothelial AJs. Further functional experiments showed that ARVCF plays a significant role in the maturation of VE-cadherin-based AJs: ARVCF associates with a selective pool of VE-cadherin molecules that are unbound to p120-catenin through a mechanism involving the intrinsically disordered regions (IDRs) of ARVCF. The direct association of ARVCF to VE-cadherin is important for junction stabilization. The existence of two types of VE-cadherin pools, one bound to p120-catenin and one bound to ARVCF, and the notion that ARVCF binds to E-cadherin in epithelial cells,^46^ indicate that the contribution of ARVCF to junction strengthening is conserved for classical cadherins. Together, our results present ARVCF at an essential position in the AJ complex where it regulates junction maturation to safeguard endothelial barrier integrity.

The core VE-cadherin interactome contains several catenins, all of which are members of the armadillo-domain protein family to which ARVCF also belongs. Within this family, ARVCF shares the greatest structural similarities with p120-catenin,^31^ a well-characterized cadherin interactor in a multitude of cell types.^47^^,^^48^ ARVCF has been reported to bind to E-cadherin via the same domain through which p120-catenin binds.^49^^,^^50^ Our study showed enhanced interaction between ARVCF and VE-cadherin at junctions as endothelial monolayers matured, while the association between VE-cadherin and p120-catenin was found to remain stable during this process. Furthermore, we found that ARVCF binds mutually exclusively to unique pools of VE-cadherin that are unbound to p120-catenin, supporting the idea of distinct or sequential roles for these proteins. This is consistent with earlier work in epithelial cells where ARVCF was reported to compete with p120-catenin for binding to E-cadherin through their central armadillo repeats.^46^^,^^49^^,^^50^ We found that the C-terminal IDRs of ARVCF support binding to VE-cadherin. Such regions lack a stable, well-defined, three-dimensional structure and are known for their scaffolding potential.^51^^,^^52^ Intriguingly, ARVCF IDRs are sequentially distinct from the protein domain in p120-catenin,^53^ pointing to a specialized function of ARVCF at AJs.

The increased junctional association of ARVCF during junction maturation indicates that ARVCF contributes to the assembly or stabilization of cadherin-based complexes. The formation of tightly packed cadherin clusters confers strong adhesion between cells.^54^ We found that the genetic depletion of *ARVCF* results in gaps in endothelial monolayers. This result can be explained by a potential loss of cadherin clustering or disruption of avidity.^55^^,^^56^ These observations support a model where ARVCF maintains strong cell-cell adhesions by facilitating cadherin clustering.

An alternative mechanism of how ARVCF supports junction maturation may involve the ARVCF IDRs in the coupling of distinct adhesion systems. In epithelial cells, ARVCF has been reported to interact with both E-cadherin and the tight junction proteins ZO-1 and ZO-2.^37^ Notably, ARVCF appears at early contact sites where the formation of both junction types begins, suggesting that it may participate in coordinating the spatial or temporal assembly of AJs and tight junctions. In endothelium, tight junction organization follows AJ formation.^4^^,^^57^^,^^58^ The presence of ARVCF at sites of early adhesion, and its known interactions with both junction types, raise the possibility that it contributes to junctional crosstalk during cell-cell contact maturation. Additionally, the conformational flexibility of ARVCF terminal IDRs may facilitate homotypic interactions, potentially creating a feedback loop that promotes its junctional enrichment. A similar function has been described for the N-terminal disordered region of the β-catenin-related protein WRM-1 in *C. elegans*, which is required for self-association.^59^ Such IDR features point to a role for ARVCF as a tunable factor in junctional stabilization.

Given that ARVCF appears to function primarily during the maturation of endothelial junctions, we do not assume that its junctional interaction is directly controlled by permeability-inducing factors. The mass spectrometry analysis demonstrated that ARVCF can bind to VE-cadherin in the absence of phosphorylation; however, we do not exclude the possibility that this interaction is maintained under phosphorylated conditions. Notably, under standard culture conditions, phosphorylation at Y731 is already high, yet ARVCF binding remains strong. In addition, ARVCF still co-localizes with VE-cadherin in thrombin-treated endothelial cells, a condition in which VE-cadherin Y685 phosphorylation levels are elevated.^23^

Endothelial migration and junction remodeling are inherently force regulated.^16^^,^^60^^,^^61^^,^^62^ The importance of ARVCF for these processes suggests that it contributes to junctional mechanotransduction. In the mouse lens, ARVCF is required for maintaining normal biomechanical properties; its absence leads to lens stiffening and ultimately cataract formation.^36^ Interestingly, *Arvcf* depletion resulted in reduced membrane localization of N-cadherin, β-catenin, and α-catenin in the lens epithelia, an effect we did not observe in ECs. These cell types differ in the relative expression levels of junctional proteins: ARVCF is markedly more abundant than p120-catenin in lens tissue, in contrast to other connecting tissues, including endothelium, where p120-catenin typically predominates. p120-catenin is necessary to retain (V)E-cadherin at the membrane.^49^^,^^63^ In ECs, the pool of p120-catenin-bound VE-cadherin suffices to maintain surface levels in the absence of ARVCF. By contrast, in xenopus, surface levels of C-cadherin were found to be reduced after depleting both ARVCF and p120-catenin,^64^ indicating partial functional compensation between the two. ARVCF and p120-catenin likely act as context-dependent regulators of VE-cadherin-mediated adhesion, with their relative abundance and recruitment contributing to junctional resilience in various mechanical environments within the vasculature. In summary, our study points to a reinforcing role for ARVCF in endothelial junction maturation and stability.

### Limitations of the study

A limitation of this study is that the overexpression of fluorescently tagged proteins may not fully recapitulate the dynamics or biochemical properties of their endogenous counterparts. In addition, lentiviral-mediated knockdown approaches may not result in complete depletion of target proteins, which could lead to an underestimation of ARVCF function in endothelial cells. Finally, the imaging approaches used here are limited in spatial resolution, and the application of super-resolution microscopy in future studies may provide deeper insight into the mechanisms underlying ARVCF recruitment to cell-cell junctions.

## Resource availability

### Lead contact

Requests for further information and resources should be directed to and will be fulfilled by the lead contact, Dr. Stephan Huveneers, (s.huveneers@amsterdamumc.nl).

### Materials availability

Materials generated and used in this study are available upon request to the lead contact.

### Data and code availability

• Mass spectrometry proteomics data are available via ProteomeXchange with identifier: PXD068012.
• This paper does not report original code.
• Any additional information required to reanalyze the data reported in this paper is available from the lead contact upon request.

## Acknowledgments

This work was financially supported by the Netherlands Organization of Scientific Research (10.13039/501100001826ZonMw Vidi grant 016.156.327 and 10.13039/501100001826ZonMw Vici grant 09150182310041) and a grant from the 10.13039/501100023406Rembrandt Institute for Cardiovascular Sciences. Graphical abstract was created with Biorender.com.

## Author contributions

Conceptualization, R.M.S., T.S.v.K.-M., and S.H.; data curation, A.J.H.; formal analysis, R.M.S., I.d.H., A.J.H., and M.v.d.B.; funding acquisition, S.H.; investigation, R.M.S., T.S.v.K.-M., I.d.H., A.J.H., F.P.J.v.A., A.d.H., A.-M.D.v.S., S.T., and S.H.; methodology, R.M.S.; supervision, J.D.v.B., M.v.d.B., and S.H.; validation, R.M.S. and I.d.H.; visualization, R.M.S., T.S.v.K.-M., and S.H.; writing – original draft, R.M.S., T.S.v.K.-M., and S.H.; writing – review & editing, R.M.S., J.D.v.B., M.v.d.B, and S.H.

## Declaration of interests

The authors declare no competing interests.

## STAR★Methods

### Key resources table

**Table:** 

REAGENT or RESOURCE	SOURCE	IDENTIFIER
**Antibodies**		

Rabbit anti-ARVCF	Thermo Fisher Scientific	PA5-64129; RRID:AB_2638256
Rabbit anti-ARVCF	Abcam	ab172633
Mouse anti-GFP	Santa Cruz	SC-9996; RRID:AB_627695
Rabbit anti-VE-cadherin	Cell Signaling Technology	D87F2; RRID:AB_10839118
Mouse anti-VE-cadherin	BD Biosciences	610252; RRID:AB_2276073
Mouse anti-p120-catenin	BD Biosciences	610134; RRID:AB_397537
Rabbit anti-β-catenin	Sigma Aldrich	C2206; RRID:AB_476831
Rabbit anti-β-actin	Cell Signaling Technology	4967; RRID:AB_330288
anti-rabbit-HRP	Bio-Rad	170–6515; RRID:AB_11125142
anti-mouse-HRP	Bio-Rad	172–1011; RRID:AB_11125936
mouse anti-α-catenin	Thermo Fisher Scientific	13–9700; RRID:AB_2533044
mouse anti-β-catenin	BD Biosciences	610153; RRID:AB_397554
mouse anti-CD31 (monoclonal IgG1κ purified in-house from hybridoma)	N/A	N/A
Mouse anti-GM130	BD Biosciences	610823; RRID:AB_398142
anti-mouse AlexaFluor (AF) AF488, AF594, AF647	Thermo Fisher Scientific	A-21200; RRID:AB_2535786, A-21201; RRID:AB_2535787, A-21463; RRID:AB_2535869
anti-rabbit AlexaFluor AF488, AF594, AF647	Thermo Fisher Scientific	A-21441; RRID:AB_2535859, A-21442; RRID:AB_2535860, A-21443; RRID:AB_2535861
anti-mouse IgG-APC	Thermo Fisher Scientific	31981; RRID:AB_429724

**Chemicals, peptides, and recombinant proteins**		

Endothelial growth medium 2 kit	Promocell	C-22211 + C-39216
Penicillin-Streptomycin	Gibco	15140122
Fibronectin	Sigma Aldrich	F0895
Dulbecco’s Modified Eagle Medium	Gibco	11320033
Fetal Calf Serum	Gibco	10270106
Gelatin	Sigma Aldrich	G7041
*Trans*-IT-LT1	Mirus	MIR 2300
Puromycin	Sigma Aldrich	P8833
PBS	Sigma Aldrich	D8537
EDTA	Sigma Aldrich	E7658
Protease inhibitor	Thermo Fisher	A32955
Phosphatase inhibitor	Roche	4906845001
NP-40	Sigma Aldrich	492018
Urea	Life Technologies	AM9902
Dithiothreitol	Thermo Scientific	R0861
Iodoacetamide	Thermo Scientific	122271000
MS-grade trypsin	Promega	V5280
Acetic acid	Sigma Aldrich	695092
Acetonitrile	BioSolve	012078
Trifluoracetic acid	Thermo Scientific	A12198
Laemmli reduced sample buffer with 4% β-mercaptoethanol	Sigma Aldrich	S3401
Bovine Serum Albumin	Sigma Aldrich	A8806
Tris-buffered saline	Sigma Aldrich	T5912
Enhanced chemiluminescence detection	Thermo Fisher Scientific	34580
PFA	Merck	30525-89-4
Triton X-100	Sigma Aldrich	X100
Mowiol	Sigma Aldrich	81381
DABCO	Sigma Aldrich	290734
ProLong Diamond Antifade	Thermo Fisher Scientific	P36961
EZ-link biotin	Thermo Fisher Scientific	20217
AF488-conjugated streptavidin	Thermo Fisher Scientific	S11223
L-cysteine	Sigma Aldrich	W326305
TriplE	Gibco	12563011
Dimethyl sulfoxide (DMSO)	Sigma Aldrich	472301

**Critical commercial assays**		

Magnetic GFP-trap agarose beads	Chromotek	gtma-20
Orbitrap Fusion mass spectrometer	Thermo Fisher Scientific	N/A
4-12% gradient SDS-PAGE gels	Thermo Fisher Scientific	NW042125BOX
Amersham ImageQuant 800 GxP	Cytiva	29653452
ibidi μ-slides VI^0.4^	Ibidi	80666
ECIS ZTheta machine	Applied BioPhysics	N/A
ECIS gold electrodes 8w10E+	Applied BioPhysics	72040
CytoFLEX flow cytometer	Beckman Coulter	N/A

**Deposited data**		

Mass spectrometry proteomics data	ProteomeXchange	PXD068012

**Experimental models: Cell lines**		

Pooled human umbilical vein endothelial cells (HUVEC)s	Lonza	P1052, C2519A
Pooled human arterial endothelial cells (HAEC)s	Lonza	CC2535
Lenti-X HEK293T cells	Takara	632180

**Oligonucleotides**		

VE-cadherin Y658F forward primer	GAGATGGACACCACCAGCTT CGATGTGTCGGTGCTCAAC	This study
VE-cadherin Y658F reverse primer	GTTGAGCACCGACACATCG AAGCTGGTGGTGTCCATCTC	This study
VE-cadherin Y685F forward primer	GCCCGGCCTTCCCTCTTTG CGCAGGTGCAGAAG	This study
VE-cadherin Y685F reverse primer	CTTCTGCACCTGCGCAAA GAGGGAAGGCCGGGC	This study
VE-cadherin Y731F forward primer	CGACACGCTGCACATCTTC GGCTACGAGGGCTCC	This study
VE-cadherin Y731F reverse primer	GGAGCCCTCGTAGCCGAAG ATGTGCAGCGTGTCG	This study

**Recombinant DNA**		

pLV-hVE-cadherin-GFP	Dorland et al.^65^	N/A
pLV-hp120-catenin-mCherry	Huveneers et al.^16^	N/A
pLV-hVE-cadherin-GFP (Y658F)	This study	N/A
pLV-hVE-cadherin-GFP (Y685F)	This study	N/A
pLV-hVE-cadherin-GFP (Y731F)	This study	N/A
pLV-CMV-MCS-IRES-puro-SIN	Addgene	N/A
pLV-GFP-hARVCF (long isoform 1)	This study	N/A
pEGFP-hARVCF(FL)	Rappe et al.^66^	N/A
pEGFP-hARVCF(NA)	Rappe et al.^66^	N/A
scrambled shRNA	Sigma MISSION® library	SHC002
shARVCF#1 (TRCN0000123310)	Sigma MISSION® library	TRCN0000123310
shARVCF#2 (TRCN0000123313)	Sigma MISSION® library	TRCN0000123313

**Software and algorithms**		

MaxQuant 1.6.2.10	Tyanova et al.^67^	−
UniProt human protein database	UniProt consortium	https://www.uniprot.org
R statistical computing environment	R Foundation for Statistical Computing	https://www.r-project.org
LIMMA R package	Bioconductor	https://bioconductor.org/packages/limma
NIS-Elements AR	Nikon Instruments	RRIS:SCR_014329
ECIS ZTheta software	Applied BioPhysics	https://www.biophysics.com
FlowJo	BD Biosciences	https://www.flowjo.com
FIJI	Schindelin et al.^68^	https://imageJ.net/Fiji
Gel Analyzer plugin (FIJI)	ImageJ	https://imagej.net
Coloc2 plugin (FIJI)	ImageJ	https://imagej.net/plugins/coloc-2
TrackMate plugin (FIJI)	ImageJ	https://imagej.net/plugins/trackmate
GraphPad Prism v10.2	GraphPad Software	https://www.graphpad.com
Adobe Illustrator	Adobe Inc.	https://www.adobe.com/products/illustrator.html
BioRender	BioRender	https://biorender.com

### Experimental model and study participant details

#### Cell culture

All cells were maintained in standard humidified incubators set to 37°C with 5% CO_2_. Pooled human umbilical vein endothelial cells (HUVECs; mix of female and male donors, P1052, #C2519A, Lonza) and human arterial endothelial cells (HAECs; male donor, #CC2535, Lonza) were cultured until at most passage 8 in endothelial growth medium supplemented with endothelial growth factor-FCS mix (EGM2; #C-22211 + #C-39216, Promocell), 100 U/mL penicillin, and 100 μg/mL streptomycin (#15140122, Gibco) on fibronectin (FN) coated surfaces. Endothelial cells tested negative for mycoplasma and were authenticated by immunostaining for VE-cadherin. Lenti-X HEK293T cells (#632180, Takara) were cultured in DMEM (#11320033, Gibco) + 10% FCS (#10270106, Gibco).

#### Lentiviral transduction

Lenti-X HEK293T cells were used to produce lentivirus by transfecting gene plasmids together with third generation packaging plasmids^69^ and using *Trans*-IT-LT1 (MIR 2300, Mirus) as described previously.^70^ HUVECs were transduced at 60% confluence by overnight incubation with the lentiviral particles and subsequently selected overnight with 250 ng/mL puromycin (#P8833, Sigma). Selected HUVECs were analyzed at least 72h after transduction to ensure knockdown efficiency.

### Method details

#### Plasmids and cloning

The pLV-hVE-cadherin-GFP and pLV-hp120-catenin-mCherry constructs were described before.^16^^,^^65^ The three VE-cadherin[Y > F] mutations were generated through site directed mutagenesis in a pEGFP-hVE-cadherin-GFP. The [Y658F] mutation was generated using primers 5′-GAGATGGACACCACCAGCTTCGATGTGTCGGTGCTCAAC-3′ and 5′-GTTGAGCACCGACACATCGAAGCTGGTGGTGTCCATCTC-3’. The [Y685F] mutation was generated using primers 5′-GCCCGGCCTTCCCTCTTTGCGCAGGTGCAGAAG-3′ and 5′-CTTCTGCACCTGCGCAAAGAGGGAAGGCCGGGC-3’. The [Y731F] mutation was generated using primers 5′-CGACACGCTGCACATCTTCGGCTACGAGGGCTCC-3′ and 5′-GGAGCCCTCGTAGCCGAAGATGTGCAGCGTGTCG-3’. Resulting plasmids were digested with restriction enzymes SnaBI and XbaI and inserts were ligated into a pLV-CMV-MCS-IRES-puro-SIN vector using enzymes SnaBI and NheI. A GFP-human ARVCF (long isoform 1, 962 amino acids (UniProt O00192-1)) cDNA within pLV plasmid was obtained from VectorBuilder (VB230406-1221, VectorBuilder). pEGFP-hARVCF plasmids (based on long isoform 1, 956 amino acids (UniProt E9PDC3)) were a kind gift from Dr. Ilse Hofmann^66^ ([FL] = full length; [NA] = N-terminus + armadillo regions). For protein knock-down we used shRNA plasmids targeting human genes (TSC2 MISSION library, Sigma) or control shRNA (SHC002; Sigma-Aldrich). To deplete ARVCF we used shRNA TRCN0000123310 as shARVCF#1; and TRCN0000123313 as shARVCF#2.

#### Co-immunoprecipitation

Co-IP on untransduced HUVECs, HUVECs expressing GFP alone or GFP-tagged proteins was performed with magnetic GFP-trap agarose beads (#gtma-20, Chromotek). Cells were washed 2 times with cold PBS supplemented with 1 mM CaCl_2_ and 0.5 mM MgCl_2_ and incubated for 10 min with freshly made ice-cold lysis buffer comprised of 10 mM Tris-HCl pH 7.5, 150 mM NaCl, 0.5 mM ethylenediaminetetraacetic acid (EDTA) (all reagents Sigma Aldrich), protease and phosphatase inhibitors (#A32955, Thermo Fisher; #4906845001, Roche), and 0.5% Nonidet P-40 substitute (NP-40, #492018, Sigma Aldrich). Cell lysates were collected in protein LoBind tubes (#0030108116, Eppendorf; used throughout this procedure), and centrifuged at 12,000 g at 4°C for 10 min. Supernatants were transferred to clean tubes and incubated at 4°C overnight with GFP nanobeads that were washed and equilibrated 3 times with PBS and 3 times with lysis buffer. The next day, immunoprecipitated beads were washed 3 times with freshly made wash buffer (lysis buffer without NP-40) followed by 2 washes with ice-cold PBS, each time transferring to clean tubes. The resulting samples were then used either for Western Blot analysis or processed for mass spectrometry analysis.

#### Mass spectrometry sample preparation

For mass spectrometry (MS) analysis, co-immunoprecipitated proteins were reduced on-bead in 1 M urea (#AM9902, Life Technologies), 10 mM dithiothreitol (#R0861, Thermo Scientific) in 100 mM Tris-HCl pH 7.5 (#17926, Life Technologies) for 20 min at 20°C and alkylated with 50 mM iodoacetamide (#122271000, Thermo Scientific) for 10 min at 20°C. Proteins were detached from the GFP nanobeads by incubation with 250 ng MS-grade trypsin (cat# V5280, Promega) for 2 h at 20°C. Beads were removed and proteins were further digested for 16 h at 20°C with 350 ng MS-grade trypsin. Tryptic peptides were desalted and concentrated using Empore-C18 StageTips (3M) and eluted with 0.5% (v/v) acetic acid (#695092, Sigma), 80% (v/v) acetonitrile (#012078, BioSolve). Sample volume was reduced by SpeedVac (Thermo Scientific) and supplemented with 2% acetonitrile, 0.1% trifluoracetic acid (#A12198, Thermo Scientific) to a final volume of 5 μL. 3 μL of each sample was injected for mass spectrometry analysis.

#### Mass spectrometry processing

Eluted peptides were separated using a reverse-phase C18 column made in-house from a Silica tip emitter (New objective, Woburn, MA, USA) filled with 1.9 μm C18 particles (Dr. Maisch, Ammerbuch-Entringen, Germany) at a flow rate of 300 nL/min with a gradient from 4% to 30% (vol/vol) acetonitrile with 0.5% acetic acid. Separated peptides were sprayed directly into a Orbitrap Fusion mass spectrometer (Thermo Fisher Scientific, Waltham, MA, USA) using a nanoflow source with a spray voltage of 2.15 kV. A Higher-energy Collisional Dissociation was performed for the most intense precursor ions selected from each 3s-duration full scan in the Orbitrap (400–1500 m/z, resolution 120,000). An isolation width of 1.6 m/z was used for the selected ions (charge ≥2). Dynamic exclusion was activated for the MS/MS scan with a repeat count of 1 and an exclusion duration of 30s.

#### Mass spectrometry analysis

The RAW mass spectrometry files were processed with the MaxQuant computational platform, 1.6.2.10.^67^ Proteins and peptides were identified using the Andromeda search engine by querying the human Uniprot database. Standard settings with the additional options match between runs, Label Free Quantification (LFQ), and unique peptides for quantification were selected. The generated ‘proteingroups.txt’ table was filtered for potential contaminants, reverse hits and ‘only identified by site’ using R. LFQ values were transformed in log2 scale and proteins were filtered for three valid values in at least one of the experimental groups. Missing values were imputed by normal distribution (width = 0.3, shift = 1.8), assuming these proteins were close to the detection limit. Statistically significant different proteins were determined using moderated T-tests using the LIMMA package in R, applying a Benjamini-Hochberg adjusted *p*-value of 0.05 and a log fold change of 1 as thresholds.

#### Western blot

Cells lysates in Laemmli reduced sample buffer with 4% β-mercaptoethanol (#444203, Sigma Aldrich) were denatured 10 min at 96°C and loaded on 4–12% gradient SDS-page gels (#NW042125BOX, Thermo Fisher Scientific) and run according to the manufacturer’s instructions. After wet or semi-dry transfer onto ethanol-activated PVDF membranes and blocking in 3% bovine serum albumin (BSA; #A8806-5G, Sigma Aldrich) or 5% milk in Tris-buffered saline (TBS; Sigma Aldrich) for at least 45 min at room temperature (RT), blots were incubated overnight at 4°C in primary antibody in TBS+blocking buffer: rabbit anti-ARVCF (#PA5-64129, Thermo Fisher Scientific or ab172633, Abcam), mouse anti-GFP (#SC-9996, Santa Cruz), rabbit anti-VE-cadherin (#D87F2, Cell Signaling Technology), mouse anti-p120-catenin (#610134, BD Biosciences), rabbit anti- β-catenin (#C2206, Sigma Aldrich), and anti-β-actin (#4967, Cell Signaling Technology); washed 3 times for 5 min in TBS; incubated 1 h at RT in secondary antibody in TBS+blocking buffer: anti-rabbit-HRP (#170–6515, Bio-Rad) or anti-mouse-HRP (#172–1011, Bio-Rad); washed another 3 times 5 min in TBS; and finally imaged using enhanced chemiluminescence detection (#34580, Thermo Fisher Scientific) on an Amersham ImageQuant 800 GxP machine (29653452, Cytiva) or by X-ray films. Protein band intensity was quantified using the FIJI/ImageJ Gel Analyzer plugin.^68^^,^^71^ Full scans of the original uncropped Western blots are included as supplemental data (Figures S4 and S5).

#### Immunofluorescence

For antibody stainings, HUVECs or HAECs were seeded at confluence and grown until desired density with medium changes every 24 h on microscope-compatible surfaces: FN-coated glass coverslips, Labtek 8-chamber imaging slides (177380, Thermo Fisher Scientific), or ibidi μ-slides VI^0.4^ (#80666, ibidi, Munich, Germany). Cells were fixed in 4% PFA in PBS++ with 1 mM CaCl_2_ and 0.5 mM MgCl_2_ for 5 min at 37°C. Fixed cells were permeabilized using 0.01% Triton (X100, Sigma Aldrich) in PBS++ for 10 min at RT, blocked in 3% BSA in PBS++ at RT for 45 min, stained with primary antibody for 1 h at RT and secondary antibodies for 45 min at RT: rabbit anti-ARVCF (#PA5-64129, Thermo Fisher Scientific), rabbit anti-VE-cadherin (#D87F2, Cell Signaling Technology), or mouse anti-VE-cadherin (#610252, BD Biosciences), mouse anti-p120-catenin (610134, BD Biosciences), mouse anti-α-catenin (13–9700, Thermo Fisher Scientific), mouse anti-β-catenin (610153, BD Biosciences), mouse anti-CD31 (monoclonal IgG1κ purified in-house from hybridoma), mouse anti-GM130 (clone 35, cat#610823, BD Biosciences), anti-mouse AlexaFluor (AF) AF488 (A-21200), AF594 (A-21201), AF647 (A-21463), anti-rabbit AF488 (A-21441), AF594 (A-21442), AF647 (A-21443) (all Thermo Fisher Scientific). Coverslips and imaging slides were mounted in Mowiol/DABCO solution or ProLong Diamond Antifade (P36961, Thermo Fisher Scientific) and stored in a dark chamber at 4°C. Cells on coverslips were washed 2 times with PBS, once with Milli-Q, and mounted in anti-fade media. Fluorescence was captured on a Nikon Eclipse TI microscope with SOLA SEII light source, 60×1.49NA Apo TIRF oil objective, standard NIKON filter cubes, using a Andor Zyla 4.2 plus sCMOS camera. Images were analyzed and prepared for inclusion in this manuscript using FIJI/ImageJ software.

#### Intensity correlation imaging analysis

Immunofluorescent images were obtained using fluorescent tag or label combinations specifically chosen to minimize overlapping excitation and emission spectra. All conditions were imaged on the same day with the same microscope settings to reduce external variation. For analysis, FIJI/ImageJ software was used to obtain junctional masks through intensity thresholding. Within masks, raw fluorescence intensity measurements per pixel across channels were analyzed using the Coloc2 plugin for FIJI/ImageJ to calculate a Pearson’s correlation coefficient using Costes’ method.^72^

#### Biotin/streptavidin permeability assay

HUVEC monolayer permeability was analyzed for control or ARVCF-depleted cells that were plated at full confluence on ibidi μ-slides VI^0.4^ (#80666, ibidi, Munich, Germany) coated with biotinylated gelatin (produced as described previously in^73^^,^^74^). After a day of confluent culture, medium was supplemented with AF488-conjugated streptavidin in a surplus stoichiometric ratio to the amount of biotin molecules. After 10 min of exposure, HUVEC monolayers were washed and immediately fixed and processed for staining with VE-cadherin antibody as described above. Biotin-bound AF488-conjugated streptavidin was analyzed using a Nikon Eclipse TI microscope with SOLA SEII light source, 60×1.49 NA Apo TIRF oil objective, standard NIKON filter cubes, using a Andor Zyla 4.2 plus sCMOS camera. FIJI/ImageJ was used to quantify GFP-positive area and intensity using the Thresholding tool, excluding areas without accompanying VE-cadherin signal, and results were corrected for cell confluence and intensity.

#### Electric cell-substrate impedance sensing

Endothelial barrier function was measured using ECIS as previously described^17^: gold electrodes (8w10E+, #72040, Applied BioPhysics) were treated with 10 mM L-cysteine (Sigma Aldrich) in PBS for 15 min at RT and coated with fibronectin for 2 h at 37°C and impedance was measured in real time for 50 h at 4000 Hz to assess junction integrity,^75^^,^^76^^,^^77^^,^^78^^,^^79^ starting immediately after seeding of 120,000 HUVECs per chamber, using the ZTheta machine (Applied BioPhysics). During measurement, cells were maintained at 37°C with 5% CO_2_. Analysis was done in GraphPad Prism.

#### Flow cytometry

VE-cadherin surface expression levels were assessed using flow cytometry. Cells were harvested with TriplE (#12563011, Gibco) and resuspended FACS buffer (PBS supplemented with 0.5% BSA and 1 mM EDTA pH 7.4–7.8, all from Sigma Aldrich). For surface receptor staining, 1∗10^5^ cells were incubated at 4°C in the dark with antibodies: first for 30 min with a surplus of mouse anti-VE-cadherin (#610252, BD Biosciences) and then, after washing, with secondary goat anti-mouse IgG-APC (#31981, Thermo Fisher Scientific). After staining, cells were washed twice with FACS buffer and resuspended in 200 μL FACS buffer for analysis. Flow cytometric acquisition was performed on a CytoFLEX flow cytometer (Beckman Coulter), and data were analyzed using FlowJo software. Isotype and single-stain controls were included for each condition.

#### Scratch wound healing

A scratch wound was introduced on confluent HUVEC monolayers grown on microscope-compatible surfaces by scraping a p200 pipette tip in a straight line across the bottom of the plate. After rinsing with EGM2, the wounded monolayers were either used for immunofluorescence or live-cell imaging. For IF imaging, monolayers were fixed 5 h after wound induction in 4% PFA in PBS++ and processed for staining as described above. For live cell imaging, cells were placed in a humidified 37°C incubated chamber (Okolab) with 5% CO_2_ on an inverted NIKON Eclipse TI microscope and phase-contrast images were captured every 15 min for 10 h using a 10x CFI Achromat DL 0.25 NA dry objective and Andor Zyla sCMOS camera. Images were analyzed and prepared for inclusion in this manuscript using ImageJ/FIJI software; scratch wound migration was analyzed using the TrackMate plugin.^80^^,^^81^

### Quantification and statistical analysis

#### Data analysis and statistics

FIJI/ImageJ was used for image analysis using the plugins mentioned above, and for image preparation for inclusion in this manuscript. Data was analyzed for statistical significance using GraphPad PRISM software. For ECIS experiments, technical duplicates were averaged per biological replicate. Western blots were corrected for background, for total protein loading, and related to control band intensity within a biological replicate condition. Statistical comparison between two groups was made using Student’s *t* test after Shapiro-Wilk normality test or Welch’s *t* test. Comparison between multiple groups was made using one-way ANOVA in combination with a D'Agostino-Pearson test for normality and the corresponding Tukey’s/Dunnett’s multiple comparisons and post-hoc tests. *p*-values are indicated in figure legends and denoted within the graph as ∗*p* < 0.05, ∗∗*p* < 0.01, ∗∗∗*p* < 0.001, ∗∗∗∗*p* < 0.0001.

## Supplemental information

Document S1. Figures S1−S5
