# Supplementary material for: VE-cadherin interaction proteomics identifies ARVCF as stabilizer of endothelial adherens junctions
Source: iScience. 2026 Mar 21;29(4):115450. doi: 10.1016/j.isci.2026.115450 (PMC13089046; doi:10.1016/j.isci.2026.115450)
Supplement: Document S1. Figures S1−S5 [file mmc1.pdf]

iScience, Volume *29*

Supplemental information

VE-cadherin interaction proteomics identifies ARVCF as stabilizer of endothelial adherens junctions

Rianne M. Schoon, Tsveta S. van Krimpen-Malinova, Iris de Heer, Arie J. Hoogendijk, Floris P.J. van Alphen, Annett de Haan, Anne-Marieke D. van Stalborch, Simon Tol, Jaap D. van Buul, Maartje van den Biggelaar, and Stephan Huveneers

Figure S1.

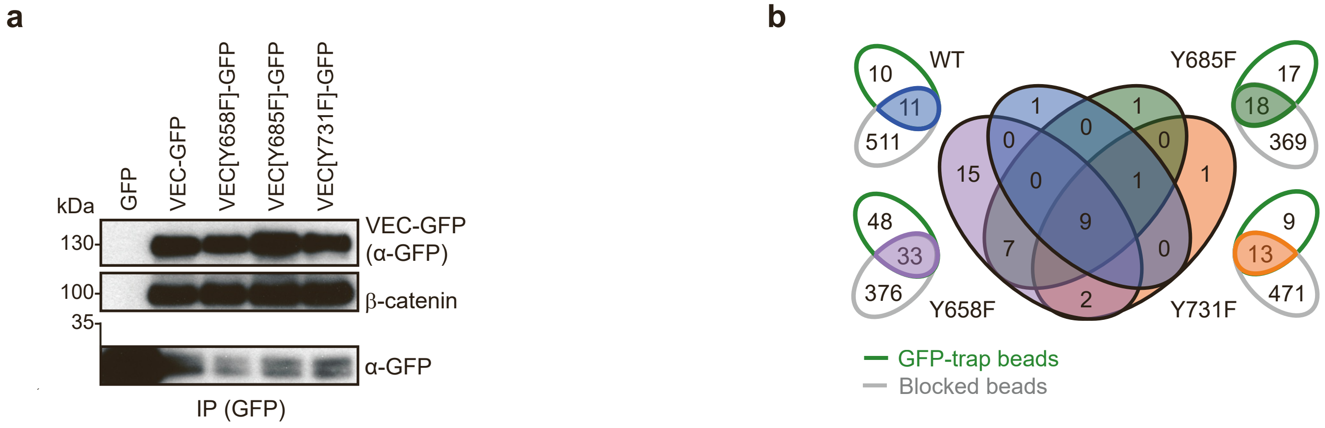

Figure S1. Identification of VE-cadherin binding proteins. a) Representative Western blot of lysates used for co-immunoprecipitation and mass-spectrometry analysis, probed for β-catenin and GFP. **b)** Venn diagram illustrating the amount of higher abundant proteins (Benjamini-Hochberg-adjusted p-value <0.05 & LOG2 FC>1 versus GFP beads and blocked beads) per VE-cadherin variant and the overlap in binding proteins between the different non-phosphorylatable [Y►F] VE-cadherin-GFP variants. Visualized using the online tool available at http://bioinformatics.psb.ugent.be/webtools/venn. Related to Figure 1.

Figure S2.

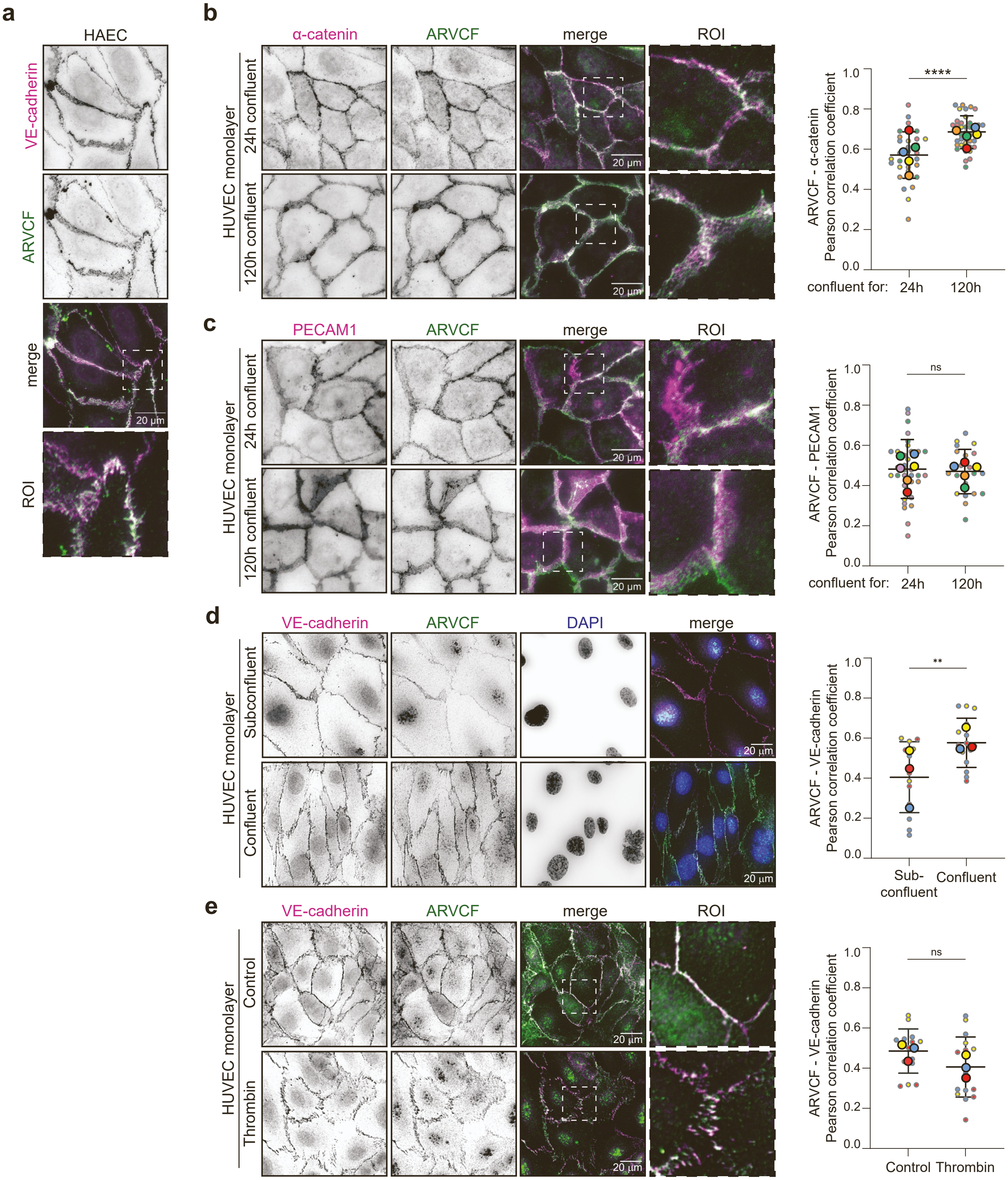

Figure S2. ARVCF localizes at VE-cadherin-based endothelial adherens junctions. a) Representative images of human arterial endothelial cells (HAECs) that were stained for endogenous ARVCF (green) and VE-cadherin (magenta). **b, c)** Representative images of human umbilical vein ECs (HUVECs) that were fixed after 24 or 120 hours at full confluence and stained for endogenous ARVCF (green) and α-catenin (b) or PECAM1 (c) in magenta. Graphs showing Pearson's correlation coefficients of pixel intensity values between ARVCF and α-catenin or PECAM1. **d)** Representative images of human umbilical vein ECs (HUVECs) at subconfluent and confluent densities 24 hours after plating, stained for endogenous ARVCF (green), VE-cadherin (magenta) and DAPI (blue). Graphs showing Pearson's correlation coefficients of pixel intensity values between ARVCF and VE-cadherin. **e)** Representative images of human umbilical vein ECs (HUVECs) that were fixed after control or thrombin treatment (1 U/ml; 10 min) and stained for endogenous ARVCF in green and VE-cadherin in magenta. Graphs showing Pearson's correlation coefficients of pixel intensity values between ARVCF and VE-cadherin, values per image (small dots) and means per independent experiment (large dots per color) ±SD. Welch's unpaired t-test, $^{**}p<0.01$, ns: non-significant. Scale bars 20 μm. Related to Figure 3.

Figure S3.

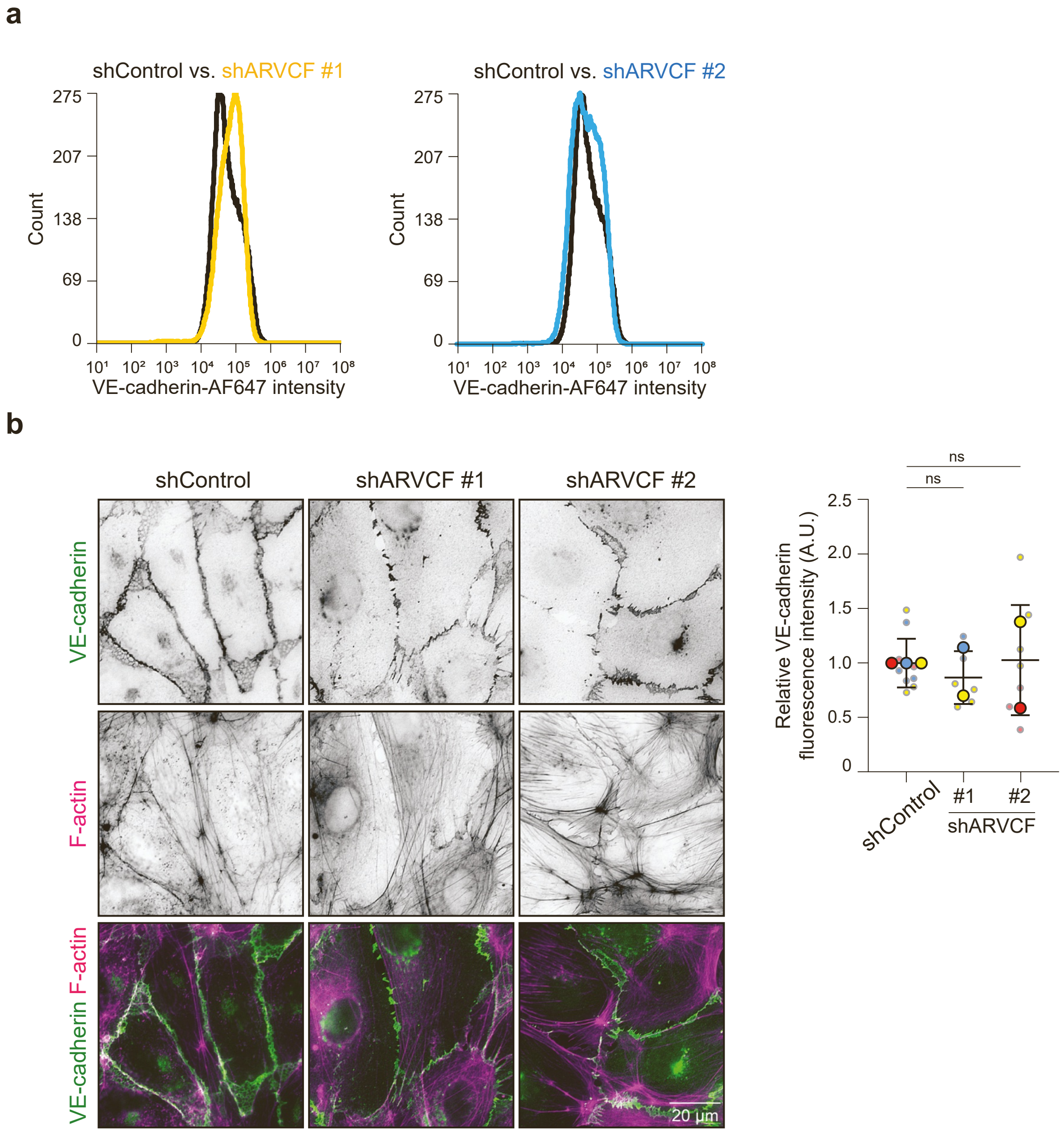

Figure S3. Characterization of shARVCF HUVECs. a) Representative graphs from fluorescence activated cell sorting analysis of VE-cadherin surface expression on HUVECs transduced with shControl or shARVCF. **b)** Representative images of HUVECs transduced with shControl or shARVCF stained for endogenous VE-cadherin (green), and F-actin (magenta). Graph showing VE-cadherin fluorescence intensity of shControl and shARVCF cells relative to control, values per image (small dots) and means per independent experiment (large dots per color) ±SD. Data from n=3 independent experiments, ANOVA with Dunnett's multiple comparison test. ns: non-significant. Scale bar 20 µm. Related to Figure 5.

Figure S4.

Fig. 2a

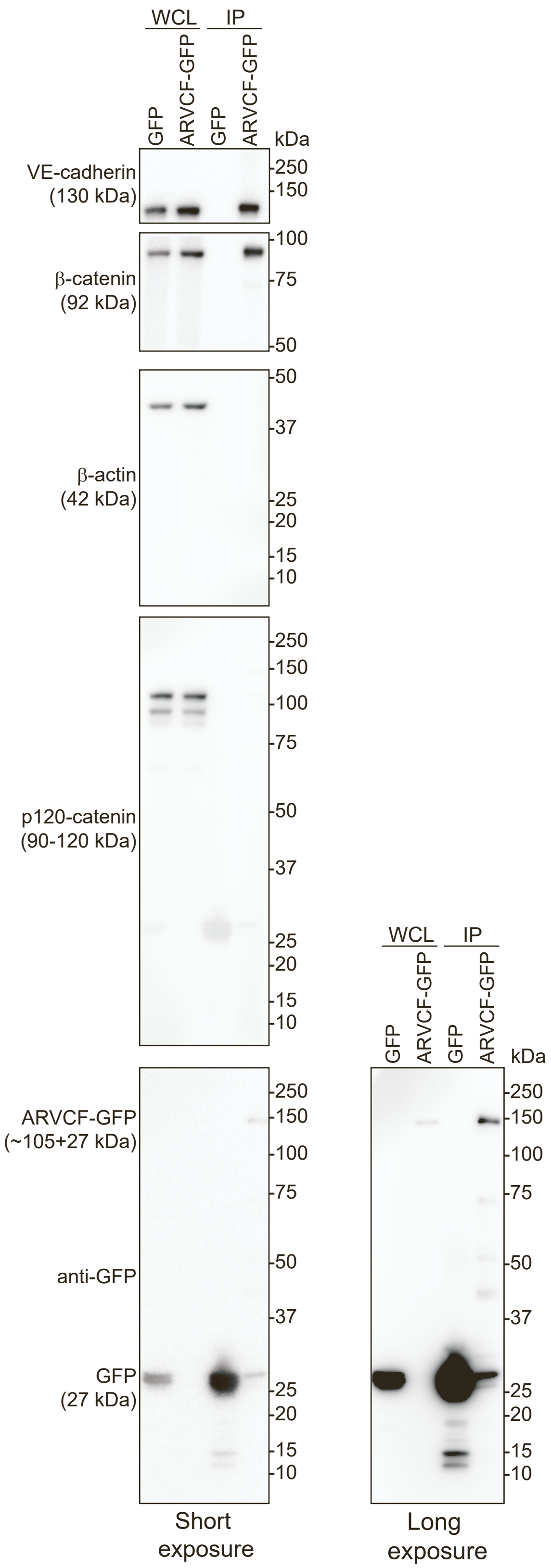

Fig. 2c

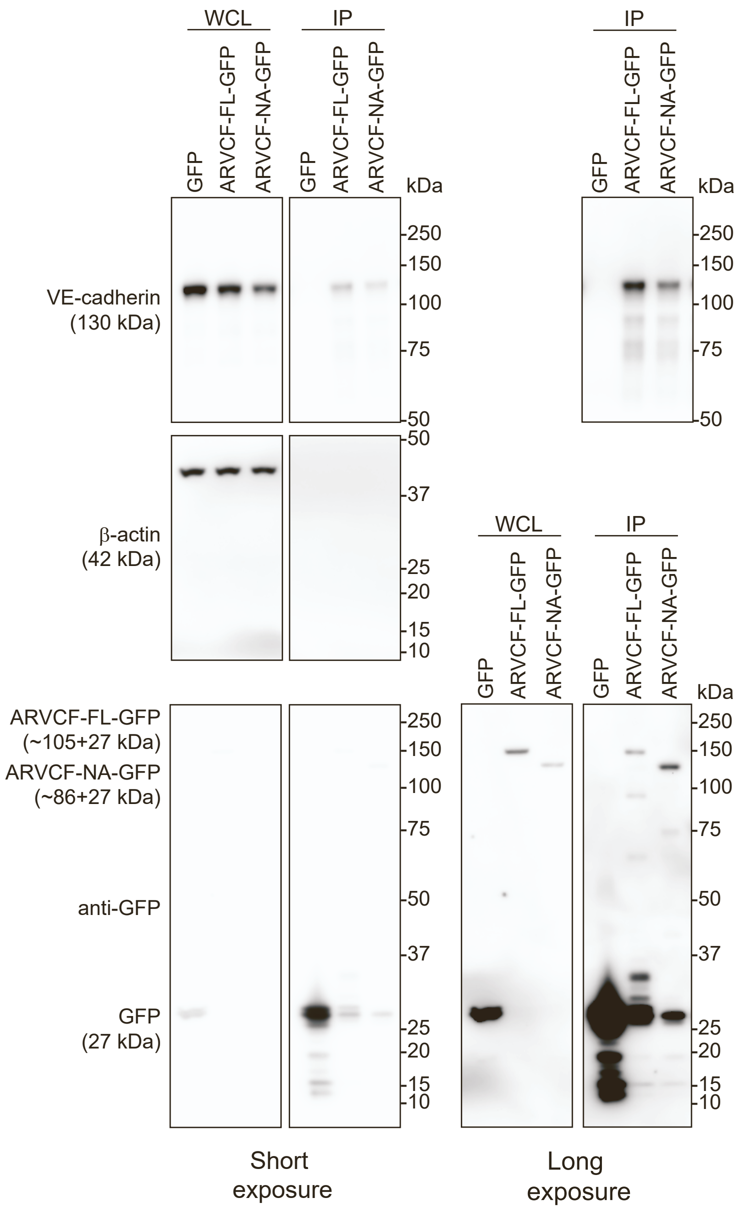

Figure S4. Full scans of the original uncropped Western blots Part I. Related to Figure 2.

Figure S5.

Fig. 2d

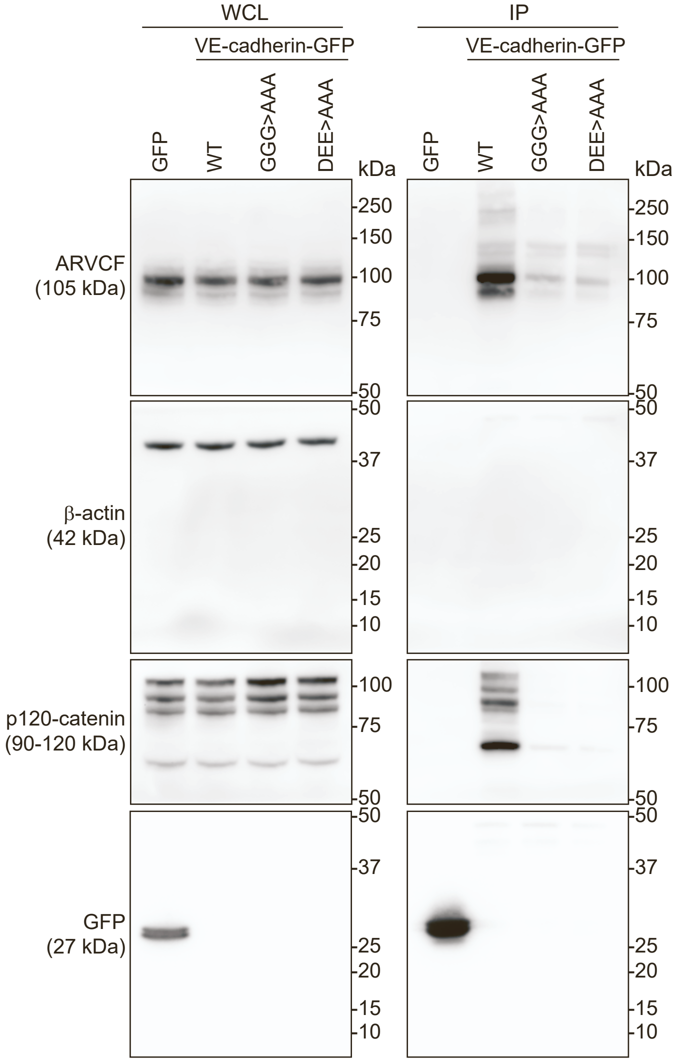

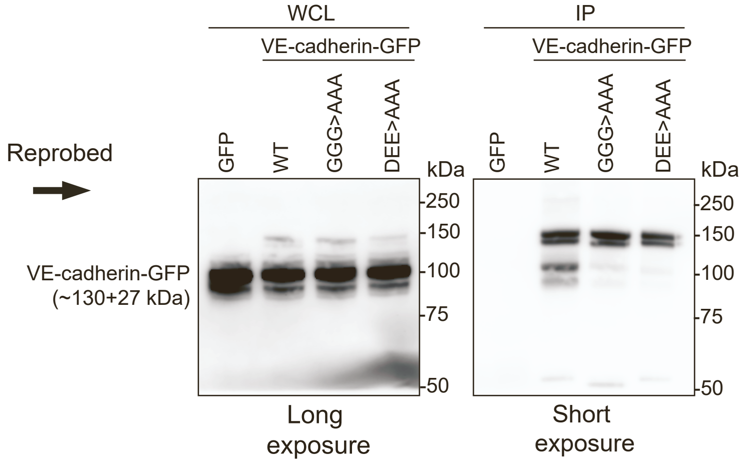

Fig. 3d

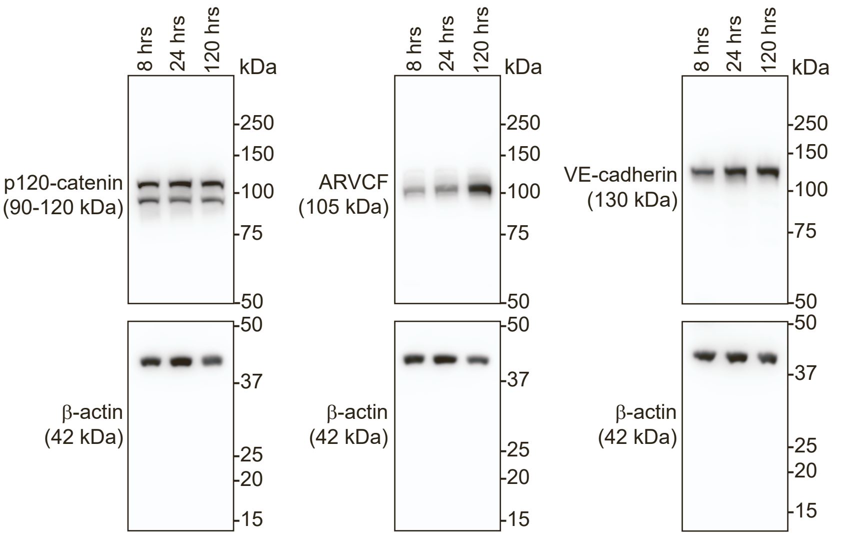

Fig. 5a

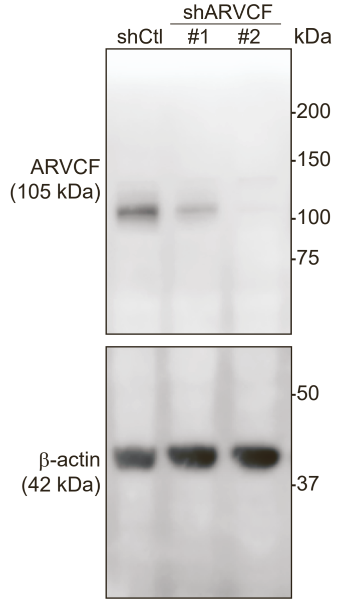

Suppl. Fig. 1a

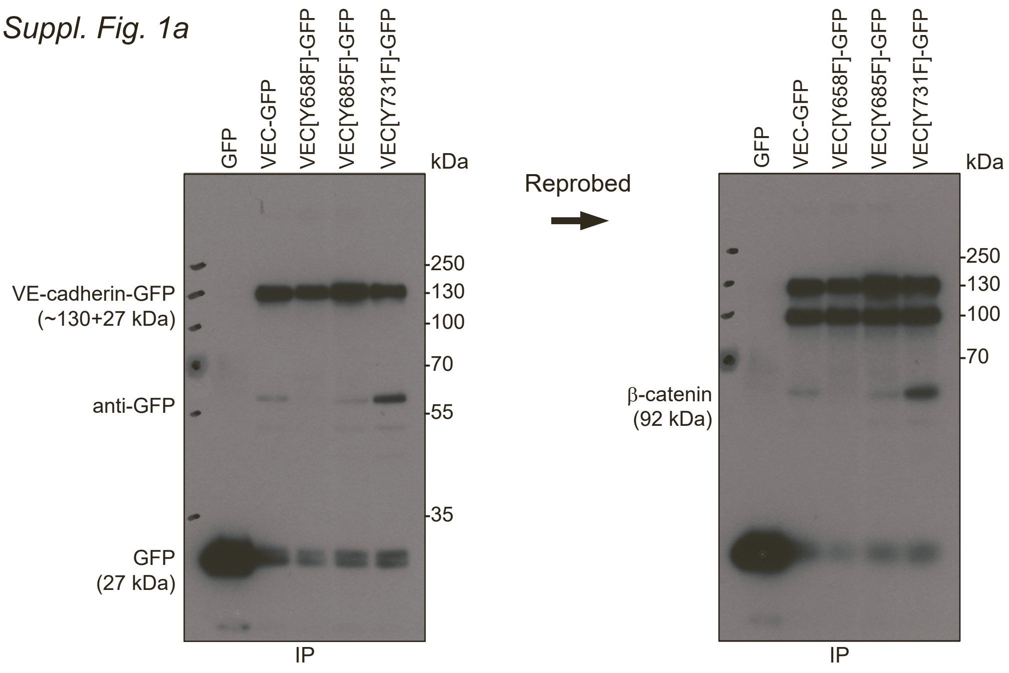

Figure S5. Full scans of the original uncropped Western blots Part II. Related to Figures 2, 3, 5 and S1.
